# Pigmentation, Melanocyte Colonization, and p53 Status in Basal Cell Carcinoma

**DOI:** 10.1155/2011/349726

**Published:** 2010-09-29

**Authors:** Lídia M. Frey, Roland Houben, Eva-B. Bröcker

**Affiliations:** Department of Dermatology, Venerology and Allergology, University of Würzburg, 97080 Würzburg, Germany

## Abstract

Basal cell carcinoma (BCC) is the most common neoplasm in the Caucasian population. Only a fraction of BCC exhibits pigmentation. Lack of melanocyte colonization has been suggested to be due to p53-inactivating mutations in the BCC cells interfering with the p53-proopiomelanocortin pathway and the production of alpha melanocyte-stimulating hormone in the tumor. To evaluate this, we determined tumor pigmentation as well as expression of melan-A and of p53 in 49 BCC tissues by means of immunohistochemistry. As expected, we observed a positive relation between tumor pigmentation and melan-A positive intra-tumoral melanocytes. Melanocyte colonization and, to a lesser extent, p53 overexpression showed intraindividual heterogeneity in larger tumors. p53 overexpression, which is indicative of p53 mutations, was not correlated to melanocyte colonization of BCC. Sequencing of exon 5–8 of the p53 gene in selected BCC cases revealed that colonization by melanocytes and BCC pigmentation is neither ablated by p53 mutations nor generally present in BCCs with wild-type p53.

## 1. Introduction

Basal cell carcinoma (BCC) of the skin is the most common neoplasm among the Caucasian population of the western world [1]. It is a low-grade keratinocyte-derived neoplasm, presents a relatively slow growth, is locally invasive and essentially destructive, with an extremely low metastatic potential. BCCs are commonly subdivided according to their histological subtypes; that is, nodular, morpheaform, micronodular, infiltrative, and superficial types that have different disease outcome [2]. A molecular hallmark of BCC is activation of the Hedgehog signalling pathway, involving mutations in Patched and Smoothened [3]. Moreover, BCC is known to be commonly associated with p53 mutations [4].

A fraction of BCC tumors exhibit pigmentation even though they are keratinocyte-derived neoplasms, due to melanocytic colonization in the tumor, as described by Florell et al. [5].

Concordance between p53 wild-type and melanocytic colonization as compared to p53-mutated tumors that lacked melanocytes in BCCs was previously described [4]. The aim of this work was to determine melanocyte colonization in a large number of pigmented and nonpigmented BCCs, and to correlate this with the p53 status. Surprisingly, we did not observe any correlation between melanocyte colonization and p53 overexpression, which is indicative of inactivating p53 mutations. Sequencing of exon 5-8 of 10 selected BCCs further confirmed that melanocyte colonization of BCC is not dependent on wild-type p53.

## 2. Materials and Methods

This study, analyzing human tumor samples was conducted according to the principles expressed in the Declaration of Helsinki. The study was approved by the Institutional Review Board of Würzburg University Hospital. All patients provided written informed consent for the collection of samples and subsequent analysis.* Tumor material, melanin staining and immunohistochemistry *paraffin-embedded blocks of previously microscopically diagnosed BCCs of the years 2007–2010 were analyzed. To increase the proportion of pigmented tumors, 9 clinically pigmented BCCs were added to a series of 40 unselected samples. Clinical data were available for all patients.

Four-micron sections were placed on poly-l-lysine-coated slides. These slides were dried at 60°C for 50 minutes followed by deparaffinization and rehydratation. The following stainings were performed: Melanin by a modified Staemmler method [6], Melan-A (clone A103, Dako, Denmark) [7], and p53 (Dako, Denmark) [8], all following routine procedures and using 3-amino-9-ethylcarbazole as chromogen.

All sections were systematically examined twice and in part by two independent observers under high-power magnification (400×). We analyzed quantitatively the immunohistochemical expression. As the positivity for melanin, melan-A, and p53 was very heterogeneous in some tumors, we selected three representative parts of each tumor and counted the number of positive cells or nuclei per field. Statistical analysis was done using the Graphpad Prism software.

**Sequence Analysis of p53 Exons 5 to 8**

Five immediately following slides from the tissue blocks were used for DNA extraction, and adjacent slides were used to confirm the presence of the lesion. Following deparaffinization, DNA was extracted using a DNA isolation kit (Qiagen, Hilden, Germany). For sequencing (SeqLab; Göttingen, Germany), the p53 exons 5, 6, 7, and 8 were amplified with BioTherm Taq polymerase (GenCraft, Münster, Germany) by seminested PCR using the following primers which all target exon flanking intron sequences:

 Exon 5-1: GTGCCCTGACTTTCAACTCTG
 Exon 5-2: ATCAGTGAGGAATCAGAGGC
 Exon 5-3: (nested primer) GGGCAACCAGCCCTGTCG
 Exon 6-1: GCCTCTGATTCCTCACTGAT
 Exon 6-2: GGAGGGCCACTGACAACCA
 Exon 6-3: (nested primer) CCAGAGACCCCAGTTGCAAAC
 Exon 7-1: AGGCGCACTGGCCTCATCTT
 Exon 7-2: AGGGGTCAGAGGCAAGCAGA
 Exon 7-3: (nested primer) TCAGAGGCAAGCAGAGGCTG
 Exon 8-1: GGACAGGTAGGACCTGATTTCCTTAC
 Exon 8-2: TGAATCTGAGGCATAACTGC
 Exon 8-3: (nested primer) TGCACCCTTGGTCTCCTCCAC

## 3. Results

Sixteen out of 49 (30.6%) BCCs analyzed in this study were clinically pigmented. The median age of the patients was 67.35 years (range 29–87 years) with a female : male ratio of 1 : 2.1. The majority (63.2%) of the BCCs were derived from the head and neck region. The most common histological subtype was the nodular subtype (32.6%). 8 of the 16 clinically pigmented BCCs were of this subtype (Table 1).

**Figure 1:**
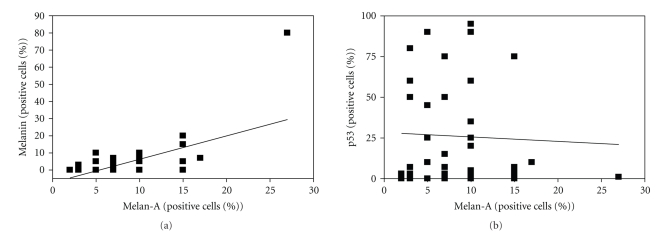
Correlation of pigmentation with melanocyte colonization (a) and lack of correlation between p53 positivity and melanocyte colonization. (b) 49 BCC tissue sections were analyzed immunohistochemically for the presence of melanin, the expression of the melanocyte marker melan-A and p53 expression. The frequency of melanin- and p53-positive tumor cells as well as the frequency of melan-A-expressing melanocytes was scored. (a) The correlation between melanocytes and pigmentation was statistically significant (*P* < .0001). (b) There was no correlation between the frequency of melanocytes and p53 expression (*P* = .77).

**Figure 2:**
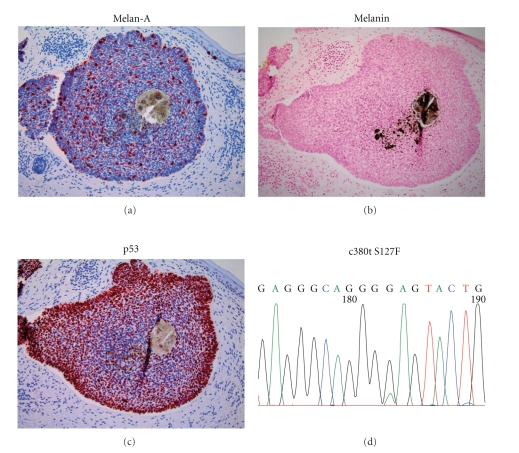
Melanocytic colonization in a BCC expressing mutant p53. Depicted are immunohistochemically stained tissue sections of a BCC displaying approximately 10% melan-A positive melanocytes (a) and high levels of nuclear p53 in 90% of the tumor cells (c). The specimen shows strong pigmentation in the centre of the lesion (b), and sequencing revealed the presence of a c380t mutation leading to an S127F amino acid exchange (d).

Surprisingly, all BCC specimens (including those lacking any detectable pigmentation) harboured at least some melan-A positive cells (Table 1). Although this might be indicative of a small population of cells which are unspecifically stained by the melan-A antibody, a highly significant (*P* < .0001) correlation between the number of melan-A positive cells and microscopic pigmentation in the melanin staining was observed (Figure 1(a)). Melanin staining and macroscopic pigmentation were highly concordant with the exception of the two cases which stained positive for melanin in the immunohistochemistry without obvious macroscopic pigmentation (Table 1). For clinically pigmented BCC, melanization occurred diffusely throughout the tumor nests (Figure 2). This is in contrast to the clinically nonpigmented BCCs, which focally showed only minimal pigmentation in the centre of the tumor mass. 

To study the relationship between melanocyte colonization and p53 status, we analyzed p53 expression since high-level expression of p53 expression is frequently regarded as an indication of p53-inactivating mutations [9]. However, the frequency of tumor cells overexpressing p53 did not correlate with the frequency of melan-A positive cells within the tumor mass (Figure 1(b)). Moreover, neither macroscopic pigmentation nor melanin expression correlated with p53 expression (data not shown). 

Since p53 immunohistologically detectable overexpression in cancer cells is not always caused by inactivating mutations [9, 10], we sequenced exon 5-8 of the p53 gene in ten selected BCC samples to confirm that the p53 expression status is indeed a measure of p53 mutation in our BCC tissues. For this purpose, we selected six tumor samples displaying macroscopic pigmentation, a relative high proportion of melanin positive cells, as well as p53 overexpression and four nonpigmented BCCs poor in melanocytes and lacking detectable p53 expression. In the latter, we observed only wild-type sequences for p53 (Table 1), suggesting that the lack of melanocyte colonization in these four tumors is indeed not due to a p53 mutation. In four pigmented BCCs, which displayed high or intermediate positivity for p53, we were able to detect p53 mutations (Table 1 and Figure 2). All four mutations detected are described as missense mutations detected in several different cancer types [11]. This confirms that p53 overexpression can be indeed indicative for the presence of p53 mutations in BCC specimens and even more importantly demonstrates that the presence of p53 mutations in BCC cells is not incompatible with melanocyte colonization. In two further pigmented BCC samples with a high and intermediate proportion of p53 overexpressing tumor cells, respectively, we could not detect a p53 mutation by sequencing of exons 5-8.

## 4. Discussion

Pigmentation of the skin results from the synthesis of melanin in the pigment-producing cells, the melanocytes, followed by distribution and transport of pigment granules to neighbouring keratinocytes [12]. Keratinocyte-derived basal cell carcinomas display pigmentation in approximately 10% of cases in the Caucasian population [13]. In our selected series, 30% of the lesions showed macroscopic pigmentation. The degree of pigmentation in the BCC samples was related to the number of melanocytes and the distribution of melanin in the tumor. However, the fact that we detected melan-A positive cells also in all melanin-negative BCCs suggests that BCC colonizing melanocytes are not activated in the means of melanin synthesis and transport in every case. It might be also indicative of a small subpopulation of cells which are unspecifically stained by the melan-A antibody. Nevertheless, the highly significant correlation between melan-A and melanin-positive cells suggests that the melan-A analysis is still a valid measure for melanocyte colonization. 

The tumor-suppressor protein p53 is a transcription factor that plays an important role in the cellular response to genotoxic stress [14]. It has been shown to directly activate transcription of numerous genes such as those that regulate cell-cycle progression, apoptotic pathways, and others [15]. Loss of function of p53 leads to aberrant cell growth and survival responses and, as such, p53 dysregulation is a major contributor in the genesis of human cancers, in particular most skin cancers which are characterized by high frequencies of p53 inactivating mutations [4].

Furthermore, besides such anticancerogenic activity, p53 in keratinocytes has been proposed to be the central transducer of the skin-tanning response by inducing proopiomelanocortin (POMC), followed by the release of the POMC cleavage product, *α* melanocyte-stimulating hormone, and subsequent recruitment and stimulation of melanocytes [4]. This, however, has been questioned by others who consider p53 as only one important coordinator (among others) but not as the main or sole regulator of pigmentation in the suntan response [16]. These authors argue that melanocytes can be stimulated via the melanocortin 1 receptor (MC1-R) even in the absence of POMC-derived melanocortins through other ligands, or an intrinsic, ligand independent activity [17, 18].

One finding of Cui and colleagues supporting the central role of keratinocytic p53 in melanocyte activation was the tight correlation they found in 23 BCCs with only the 15 p53 wild-type tumors showing melanocyte colonization while the 8 BCCs for which they detected p53-inactivating mutations lacked cells expressing the melanocytic marker MITF [4]. In our series of 49 BCC samples, we found several examples fitting into this pattern, but overall no significant correlation between p53 overexpression as an indication of p53 mutation and melanocyte colonization was found by statistical evaluation. A limitation of our (and other's) interpretation of data is the heterogeneity of pigmentation, melanocyte colonization, and p53 overexpression in an individual tumor. This was obvious in some larger nodular BCC studied by immunohistology. Therefore, for mutation analyses, we selected lesions with clear-cut homogeneous expression patterns. 

Sequencing of the p53-gene in 10 selected samples demonstrated that p53 overexpression correlates with the presence of a mutation. Most importantly, however, it confirmed that pigmentation and melanocyte colonization may well be present in p53 mutant BCC. Therefore, our data support the view that p53 is unlikely to be the sole regulator of pigmentation [16].

## Figures and Tables

**Table 1:** Clinical, microscopic, immunohistologic, and sequencing data of the basal cell carcinomas analysed in this study.

Gender	Localization	Growth pattern	Macroscopic pigmentation	Melanin	Melan-A	p53 expression	p53 sequencing
m	Nose	Micronodular	No	0%	10%	60%	
f	Nose	Morpheaform	No	0%	7%	75%	
m	Forearm	Nodular	Yes	80%	27%	1%	
m	Nose	Micronodular	Yes	20%	15%	0%	
f	Lower leg	Superficial	No	0%	5%	90%	
f	Chin	Superficial	No	3%	3%	7%	
m	Cheek	Nodular	No	0%	15%	3%	
m	Temple	Nodular	No	0%	3%	50%	
m	Neck	Nodular	No	0%	10%	5%	
m	Cheek	Infiltrative	No	0%	10%	1%	
m	Nose	Nodular	No	0%	10%	3%	
m	Forehead	Morpheaform	No	0%	2%	3%	
f	Nose	Nodular	No	0%	15%	0%	
*m*	*Breast*	*Nodular*	*No*	*0%*	*3%*	*0%*	*wt*
m	Temple	Superficial	No	0%	7%	75%	
f	Periorbital	Nodular	No	0%	10%	0%	
m	Back	Nodular	No	0%	15%	0%	
m	Shoulder	Superficial	No	0%	7%	3%	
m	Neck	Nodular	No	0%	10%	25%	
*f*	*Cheek*	*Superficial*	*No*	*0%*	*3%*	*0%*	*wt*
*m*	*Scapula*	*Nodular*	*Yes*	*15%*	*15%*	*5%*	*g605a 202H*
*m*	*Cheek*	*Nodular*	*Yes*	*10%*	*10%*	*95%*	*g743a 248A*
m	Forehead	Nodular	No	0%	3%	3%	
*m*	*Scalp*	*Superficial*	*Yes*	*7%*	*17%*	*10%*	*wt*
f	Shoulder	Infiltrative	Yes	7%	7%	0%	
m	Sternum	Infiltrative	No	0%	2%	0%	
m	Sternum	Micronodular	No	0%	3%	60%	
m	Nose	Infiltrative	No	0%	5%	25%	
*f*	*Shoulder*	*Micronodular*	*Yes*	*5%*	*7%*	*75%*	*c844g 282G*
m	Forehead	Micronodular	No	0%	5%	45%	
m	Temple	Morpheaform	No	0%	7%	50%	
f	Forehead	Morpheaform	No	0%	3%	0%	
m	Cheek	Micronodular	No	0%	15%	0%	
*f*	*Cheek*	*Morpheaform*	*No*	*0%*	*3%*	*0%*	*wt*
f	Nose	Micronodular	No	10%	5%	0%	
m	Nose	Micronodular	No	0%	10%	0%	
f	Nose	Morpheaform	No	0%	3%	80%	
m	Temple	Infiltrative	No	0%	15%	75%	
*m*	*Occipital*	*Morpheaform*	*No*	*0%*	*3%*	*0%*	*wt*
m	occipital	Morpheaform	No	0%	3%	0%	
m	Arm	Nodular	Yes	5%	15%	7%	
m	Breast	Nodular	Yes	5%	10%	20%	
*f*	*Chest*	*Superficial*	*Yes*	*7%*	*10%*	*90%*	*c380t S127F*
m	Neck	Nodular	Yes	5%	7%	15%	
*f*	*Breast*	*Superficial*	*Yes*	*5%*	*15%*	*75%*	*wt*
f	Sternum	Superficial	Yes	5%	5%	10%	
f	Hip	Superficial	Yes	10%	10%	35%	
m	Forehead	Superficial	Yes	10%	10%	95%	
m	Back	Superficial	Yes	3%	7%	7%	

m: male; f: female; Melanin, melan-A and p53 expression were assessed by immunohistochemistry; sequencing of the p53 gene was performed for the exons 4-8.
